# Blue whales increase feeding rates at fine-scale ocean features

**DOI:** 10.1098/rspb.2022.1180

**Published:** 2022-08-31

**Authors:** James A. Fahlbusch, Max F. Czapanskiy, John Calambokidis, David E. Cade, Briana Abrahms, Elliott L. Hazen, Jeremy A. Goldbogen

**Affiliations:** ^1^ Hopkins Marine Station, Department of Biology, Stanford University, Pacific Grove, CA, USA; ^2^ Cascadia Research Collective, Olympia, WA, USA; ^3^ Center for Ecosystem Sentinels, Department of Biology, University of Washington, Seattle, WA, USA; ^4^ Environmental Research Division, NOAA Southwest Fisheries Science Center, Monterey, CA, USA

**Keywords:** baleen whale, habitat resource selection, movement ecology, Lagrangian coherent structures, finite-time Lyapunov exponent, biologging

## Abstract

Marine predators face the challenge of reliably finding prey that is patchily distributed in space and time. Predators make movement decisions at multiple spatial and temporal scales, yet we have a limited understanding of how habitat selection at multiple scales translates into foraging performance. In the ocean, there is mounting evidence that submesoscale (i.e. less than 100 km) processes drive the formation of dense prey patches that should hypothetically provide feeding hot spots and increase predator foraging success. Here, we integrated environmental remote-sensing with high-resolution animal-borne biologging data to evaluate submesoscale surface current features in relation to the habitat selection and foraging performance of blue whales in the California Current System. Our study revealed a consistent functional relationship in which blue whales disproportionately foraged within dynamic aggregative submesoscale features at both the regional and feeding site scales across seasons, regions and years. Moreover, we found that blue whale feeding rates increased in areas with stronger aggregative features, suggesting that these features indicate areas of higher prey density. The use of fine-scale, dynamic features by foraging blue whales underscores the need to take these features into account when designating critical habitat and may help inform strategies to mitigate the impacts of human activities for the species.

## 1. Background

How and where consumers obtain resources has fundamental consequences for individual fitness, species distributions and community dynamics and remains a central question in ecology. Organisms make decisions at multiple hierarchically organized spatial and temporal scales [1–3]; thus inquiry at nested scales is required to fully understand organism–environment interactions. These scales include geographical range selection (first-order selection), home range selection for the area where most time is spent (second-order selection), habitat selection within a home range or highest use habitat (third-order selection) and feeding site selection where behavioural modes shift (fourth-order selection) [4]. While research in both terrestrial and marine systems has addressed habitat selection at a range of scales, we have a limited understanding of how habitat selection at these scales translates into foraging performance.

In the ocean, biophysical interactions generate heterogeneously distributed resources, also described as patchiness [5–7]. This patchiness influences primary producers' (e.g. phytoplankton) ability to flourish, which affects prey availability and distribution for higher trophic levels, from secondary consumers to top predators [8]. The ephemerality of prey patches in space and time creates a challenge for pelagic predators, who must find and detect where and when these patches occur. Their ability to do so reliably ultimately translates into fitness and survival [9,10]. There is growing evidence that submesoscale (less than 100 km) physical processes are a missing link in our understanding of the structuring of pelagic ecosystems, especially in the context of predator–prey interactions [11–13]. For example, a multi-scale study of aggregative oceanic processes found that the majority of ecosystem interactions between seabirds and their prey occurred within ephemeral hotspots at scales less than 10 km [14]. However, our understanding of these interactions is limited by our ability to contemporaneously measure ocean dynamics and predator foraging performance at comparable scales [15,16].

Blue whales (*Balaenoptera musculus*) are an ideal study species to investigate how animals find and exploit ephemeral, patchy prey across multiple scales in the ocean. Blue whales’ large body size allows researchers to use animal-borne biologging devices [17,18] equipped with sensors that detect the kinematic signatures of their stereotypical feeding style (i.e. lunge feeding, [19,20]) and their location when they surface to breathe [21]. As bulk filter feeding krill specialists, blue whales rely on patchy and often ephemeral aggregations of krill (family: Euphausiacea) [22–25], resulting in a simplified trophic system useful in the investigation of predator behaviour in a natural context [15,26]. In the Eastern North Pacific, blue whales typically migrate into the California Current System (CCS) from May to November, where high-efficiency foraging on dense krill patches builds lipid stores that fuel long-distance migrations to breeding locations at lower latitudes [27,28]. At the seasonal scale, the arrival and duration of blue whales on foraging grounds (i.e. the CCS) is somewhat predictable; however, what drives movement and foraging behaviour within this habitat at finer scales (e.g. hours to weeks) remains less understood [29–31], largely due to a gap in environmental observations at these scales.

Remote-sensing of ocean dynamics informs our understanding of the relationship between pelagic predator habitat selection and surface current features (e.g. upwelling-driven eddies, jets and fronts) in pinnipeds [3,32], sharks [33,34], turtles [35], seabirds [36,37] and cetaceans [38,39]. These surface current features reflect oceanographic processes that propagate through the water column to depths that can reach the ocean bottom [40,41] and influence prey aggregation through physical forcing [13,42]. Multi-year studies of krill in the CCS show variability in the phenology and intensity of krill hotspots, with physical forcing being a key determinant of krill distribution in the environment [43,44]. Krill also aggregate in response to environmental processes at the scale of hours to days [45], indicating that fine- and intermediate-scale processes may be ecologically relevant to blue whales. Although previous studies have used remotely sensed aggregative surface current features to predict habitat use of deep-diving balaenopterid whales in Southern California [39], Central California [46] and the Mediterranean [38], high-resolution foraging performance data over ecologically relevant timescales are needed to determine the physical processes that influence prey and thus predator movements.

Identifying ecologically important dynamic habitat features requires high-resolution contemporaneous measures of predator foraging performance and the physical processes that drive resource distribution and density [14,16,47], which has been technically challenging to address [48]. Here, we use an integrated biologging and remote-sensing approach to investigate the drivers of blue whale foraging performance. Although multiple ecological and physiological factors affect foraging performance, feeding rate is a useful proxy for a diet specialist with highly stereotyped foraging behaviour, such as the blue whale [19,28]. Furthermore, blue whales modulate feeding rates in association with prey density and distribution [22,49]. First, we use high-resolution, intermediate-duration (e.g. 2–30 days) multi-sensor tags [17,18] to measure blue whale feeding rates and locations. Second, we use hourly high-frequency (HF) radar surface current measurements to calculate a time-dependent Lagrangian modelled proxy that reflects coherent aggregative ocean transport features at submesoscales [50,51]. We hypothesize that blue whales will co-locate with these aggregative surface current features and that their feeding rates will increase in their presence. In this study, we (i) examine whether blue whales disproportionately select these features from available habitat and (ii) quantify the influence these features have on blue whale feeding rates as a metric of foraging performance.

## 2. Material and methods

### (a) Blue whale movement and feeding data

Between 2016 and 2020, we deployed 23 high-resolution digital tags on blue whales in the CCS. Of these, we used data from 10 individuals that met criteria for inclusion described below (mean deployment duration 7.1 ± 5.0 days; table 1). To assess the relationship between blue whale feeding and submesoscale environmental features, we selected deployments that sampled high-resolution data for more than 24 h, collected GPS positions that overlapped with the sampling footprint of the coastal HF Radar network for over 66% of dives and engaged in feeding behaviour. Several individuals initiated a southbound migration, exhibiting a gradual behavioural transition during the deployment [31], and were excluded from this study.

**Table 1.** Summary of tag deployments used in this study. Dives with both an associated GPS location and FTLE data represent the sample sizes for each individual in this study. Overall mean daily feeding rate reflects the number of lunges per 24 h period from midnight to midnight for complete days only. Dive-by-dive mean feeding rate excludes non-feeding dives. Kolmogorov–Smirnov (K–S) test of whether the distribution of FTLE for blue whales lies below that of background points. Deployment date (BmYYMMDD) and tag type (i.e. Wildlife Computers (TDR) and Acousonde (A)) are denoted in the deployment name.

deployment	region	animal ID	year	length (days)	dives (excursions >10 m)			feeding rate (lunges h^−1^)		K–S test statistic (*p*-val.)
					total #	with GPS	with FTLE	overall daily mean ± s.d.	dive-by-dive mean ± s.d.	
Bm160523-A20	Central CA	CRC-3308	2016	4.1	853	770	770	5.8 ± 1.9	24.1 ± 8.5	0.07 (<0.0015)
Bm160716-A20	Southern CA	CRC-3090	2016	3.9	462	335	335	15.2 ± 2.1	29.2 ± 7.5	0.31 (<0.0001)
Bm160918-A08	Southern CA	CRC-3348	2016	4.1	907	791	670	16.9 ± 2.7	25.5 ± 9.1	0.15 (<0.0001)
Bm170622-TDR12	Southern CA	CRC-0542	2017	17.7	2728	2588	2569	14.4 ± 2.8	23.2 ± 5.6	0.11 (<0.0001)
Bm170925-TDR12	Southern CA	CRC-3427	2017	4.1	505	456	255	3.5 ± 2.8	22.0 ± 9.2	0.26 (<0.0001)
Bm170926-TDR14	Southern CA	CRC-3107	2017	4.3	373	259	259	13.8 ± 0.6	25.9 ± 7.7	0.42 (<0.0001)
Bm181021-TDR14	Central CA	CRC-3520	2018	9.1	1152	908	908	8.0 ± 3.4	21.0 ± 5.4	0.23 (<0.0001)
Bm190709-TDR14	Central CA	CRC-1789	2019	8.0	731	635	633	4.4 ± 4.2	17.6 ± 6.7	0.12 (<0.0001)
Bm190710-TDR15	Northern CA	CRC-3697	2019	13.5	1502	1062	1039	10.7 ± 5.7	24.4 ± 8.8	0.16 (<0.0001)
Bm201028-TDR17	Central CA	CRC-3732	2020	2.6	392	358	353	13.8 ± 7.9	24.9 ± 8.4	0.30 (<0.0001)
total			5	71.4	9605	8162	7791	10.7 ± 3.9	23.8 ± 7.4	0.06 (<0.0001)

Tag data used in this study (table 1) were TDR10F tags from Wildlife Computers (*n* = 7) and Acousonde acoustic tags from Greeneridge Sciences (*n* = 3) [18]. All tags sampled depth, accelerometry (greater than or equal to 32 Hz) and fast-acquisition GPS. Tags were deployed from a 6–7 m rigid-hull inflatable boat using a 5 m carbon fibre pole. Tags were attached to the animal with three or four stainless steel darts that were 65 mm long with 1–2 rows of petals [17] and were later recovered via ARGOS satellite positions and VHF telemetry. Raw kinematic data were pre-processed in MATLAB (MathWorks, Inc., v2017a) using tag analysis tools described in [52] to calculate the animal's pitch, roll and an estimate of speed from the accelerometer sensor data [53]. Individual feeding events (i.e. lunges) were identified manually using stereotypical signatures in kinematic data [19]. All processed data were down-sampled to 1 Hz for analysis and subsequent processing of the blue whale kinematic and geographical movement data we conducted in R v. 4.0.0 [54].

The sampling unit for the analyses in this study was blue whale dives, identified as excursions to a depth of greater than 10 m using the tagtools package (http://www.animaltags.org). Post-dive surface duration was calculated as the time between successive dives. Raw GPS location data were filtered for unrealistic whale speeds (e.g. greater than 6 m s^−1^) using the ArgosFilter package, v. 0.62 [55]. We used linear interpolation (interpolateTime function of the move package, v. 4.0.6 [56]) to estimate locations for dives with short intervals between known GPS locations (i.e. a gap less than 15 min). Only dives with a corresponding location at the start of a dive were included in this analysis (7791 dives, table 1) and we examined the resulting dataset for systematic bias due to gaps in GPS coverage. Dives were classified as feeding if they contained at least one identified lunge, and the feeding rate (lunges h^−1^) of each dive was calculated as follows:

$$\mathrm{feeding}\;{\mathrm{rate}}_{\left({\mathrm{dive}}^{-1}\right)}=\frac{\mathrm{no}.\;\;\;{\mathrm{lunges}}_{\left({\mathrm{dive}}^{-1}\right)}}{\mathrm{dive}\;\mathrm{duration}+\mathrm{post}\;\mathrm{dive}\;\mathrm{surface}\;\mathrm{duration}}.$$

**Figure 1.**
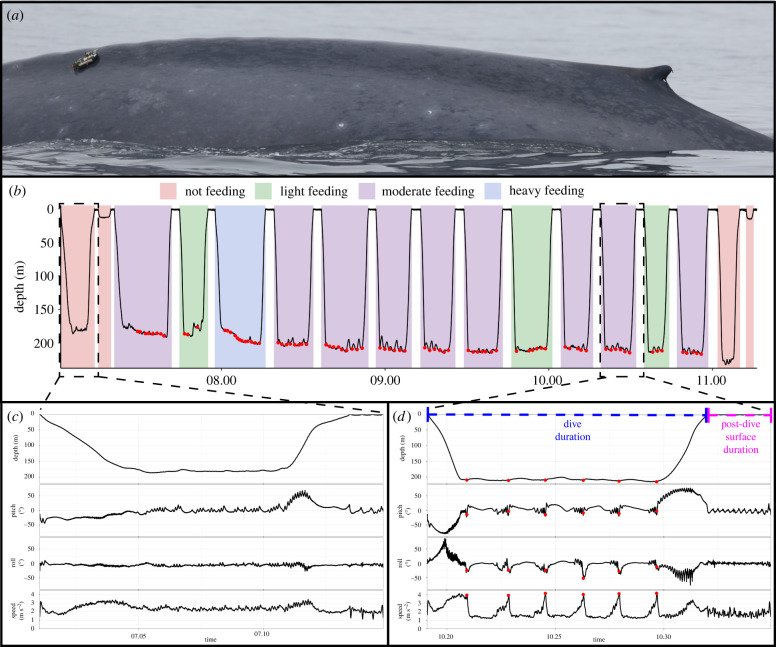
(*a*) A blue whale tagged in 2017 with a high-resolution, intermediate-duration multi-sensor tag (Wildlife Computers TRD10-F modified with a dart attachment). (*b*) Dive profile for a portion of the deployment from the blue whale in (*a*) (Bm170622-TDR12), with lunges shown by red circles and feeding states highlighted. (*c,d*) A non-feeding and feeding dive, respectively, with high-resolution data shown. Lunges (red) were identified by the stereotypical kinematic signature (e.g. changes in pitch and roll as well as a precipitous drop in speed at the time of mouth-opening); (*d*) highlights the dive duration (blue) and post-dive surface duration (magenta) used in the calculation of feeding rates.

This dive-by-dive feeding rate accounts for transit time to and from the prey at depth, the biomechanics of feeding (i.e. lunge and filter time), as well as the surface recovery period to account for the physiological constraints of diving (figure 1), and aligns with feeding rate calculations for blue whales in other studies of dive-scale foraging behaviour [22,46,57]. The foraging performance of a diving animal is optimized when the energy gain (in this case, lunge count) is maximized relative to the dive cycle duration, which includes both the time underwater and the time at the surface following a dive [58–60].

### (b) Environmental data

#### (i) High-frequency radar data

HF radar provides continuous, high-resolution measurements of ocean circulation and structure at fine and intermediate scales in coastal regions in near real time [61,62]. The technology uses terrestrial-based radar antennas to transmit electromagnetic signals and measure the backscatter as the signals are reflected off the ocean's surface. When two or more radar antennas monitor the same area, the total surface current vector (i.e. speed and direction) can be resolved [63]. Surface current vectors derived from HF radar have been validated by moored current meters [64] and other methods. The U.S. Integrated Ocean Observing System High Frequency Radar Network (IOOS HFRNet, [65]) maintains a network of HF radar monitoring stations along the US West coast, providing surface current vectors at hourly, 6 km resolution. For each deployment, surface current data were downloaded from IOOS HFRNet (http://cordc.ucsd.edu/projects/hfrnet/) for the deployment period ±7 days with a bounding box of ±1 degree around the extent of the locations for each deployment. Data gaps in the raw HF radar surface current measurements were restored using the algorithms described in Ameli and Shadden [66], selecting a concave hull (alpha shape radius of 10 km) with the detection and exclusion of land.

#### (ii) Lagrangian feature identification

**Figure 2.**
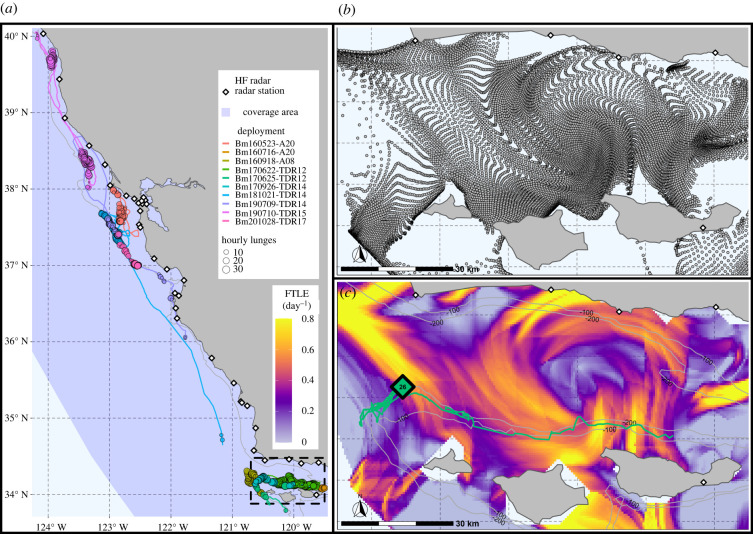
(*a*) Deployment tracks and hourly feeding summaries for 10 blue whales along the California coast, showing the HF radar coverage footprint and receiver locations (200 m isobaths shown in grey). (*b*) Locations of simulated particle tracers for 28 September 2017 14.00 (local time) after a 48 h integration (particle tracers initially seeded in an evenly spaced grid). (*c*) FTLE was calculated from the particle trajectories in (*b*), with the track from a 4-day blue whale deployment shown in green (grey 100 and 200 m isobaths). Black diamond represents the mean hourly location and number of lunges for the specific hour of FTLE data shown. (Black dashed area in (*a*) represents the spatial extent of (*b*,*c*).)

Surface current vectors alone may not adequately identify submesoscale features, such as fronts and eddies, due to the time-dependent nature of processes in the marine environment [67,68]. Instead, to characterize the strength and persistence of these features, we use a Lagrangian (i.e. time dependent) approach to identify spatially and temporally coherent transport structures (i.e. Lagrangian coherent structures, LCS). Using a Lagrangian analysis tool (Trajectory Reconstruction and Analysis for Coherent Structure Evaluation, TRACE, http://transport.me.berkeley.edu/trace/) that follows the methodology described in Shadden *et al*. [69,70], we calculated the backward-in-time finite-time Lyapunov exponent (hereafter FTLE, figure 2*b,c*), which is a scalar measure of particle aggregation and an indicator of LCS [71]. TRACE calculates FTLE by seeding the empirical surface current data with simulated tracer particles and integrating their movement trajectories over a fixed time-period, with positive FTLE values indicating locations where tracers aggregated at the end of the simulation. Ridges of elevated FTLE values identify attracting LCS [72], which represent barriers to transport that can propagate through the water column, such as fronts and eddies [73], and have biological significance to top predators [74,75]. FTLE derived from HF radar data has been shown to correlate to the movements of drifters in Monterey Bay, and the method is robust to noise and periodic measurement inaccuracies [70].

Using TRACE, we derived hourly, 600 m resolution FTLE fields from the restored hourly, 6 km resolution surface current vector fields, integrated over the preceding 48 h. We seeded the simulation with tracers at 10 × the spatial resolution of the surface current vectors, i.e. 600 m (*sensu* [70]) and applied a free-slip boundary condition to tracers near land [76], which allows the tracers to slide along the land boundary. Tracer advection used a bilinear spatial interpolation and an adaptive fourth-order Runge–Kutta–Fehlberg integration method. We selected a 48 h integration period to minimize the influence of tidal currents and reduce the chance of simulated particles exiting the domain within the integration period [73]; however, the location of LCS is generally insensitive to variations in integration length [70]. Hourly FTLE fields were derived from the preceding 48 h integration period, i.e. backward-in-time FTLE. For each blue whale dive, we extracted the FTLE value from the closest grid cell in time and space using the raster package [77] in R. Therefore, every dive is associated with a co-located FTLE value at 600 m resolution within 30 min of the dive timestamp.

### (c) Statistical analyses

The movements of free-ranging predators can be described in terms of habitat utilization in which the predator chooses a subset of the habitat available. The utilization is considered selective when a predator targets a certain set of features disproportionately to their availability in the environment [4]. The ability to identify individual blue whale feeding events from tag data provides a valuable study system well suited for evaluating higher order selection of habitat within their home range (third order) and selection of foraging sites (fourth order). We used a hierarchical approach in our analysis first to determine blue whale habitat selection at the regional scale, then assessed the modulation of feeding behaviour within this habitat. All statistical analyses were conducted in R v. 3.5.1 [54].

#### (i) Regional (third order) habitat selection

To simulate a distribution of FTLE values that an individual could have encountered in its surrounding environment (i.e. background points), random locations were drawn from the regional spatial domain for each individual (i.e. ±1 degree bounding box around the extent of the locations for each individual). Locations were generated at a 10 : 1 ratio of background locations to real locations [78], and we extracted the FTLE value of the background locations for the time-periods of their corresponding animal locations. We calculated an individually weighted mean to account for the deployment of varying lengths from the distribution of FTLE values for both real and background locations:

$${\mathrm{Weight}}_{\mathrm{individual}}=\frac{1}{{\mathrm{no}.\;\mathrm{locations}}_{\mathrm{individual}}\times \mathrm{no}.\;\mathrm{deployments}}$$

To determine whether blue whales in our study selected habitat within their regional spatial domain, we used non-parametric, two-sample Kolmogorov–Smirnov tests to determine whether FTLE values for blue whale locations are significantly greater than the surrounding area. These distribution tests illustrate habitat selection within the foraging grounds, which aligns with the third-order process (i.e. selection of habitat areas within the home or subpopulation range) from [4].

#### (ii) Feeding site (fourth order) selection

To explore blue whale feeding site selection, we used the presence or absence of feeding behaviour for each dive to examine the probability of feeding across a range of FTLE values. We fit a generalized linear mixed model (GLMM) via penalized quasi-likelihood (glmmPQL function of the MASS package, v. 7.3-53 [79]) with individuals included as a random effect to account for individual variation. We incorporated a continuous-time first-order autocorrelation structure (corCAR1, nmle package v. 3.1-147 [80]) with time since the start of the deployment as a covariate to account for serial autocorrelation in the tag data [81]. To facilitate hypothesis testing in assessing feeding site selection, we generated null model datasets that control for either spatial or temporal variation [82]. All null model analyses followed the same procedures as the blue whale data to facilitate comparison, using a GLMM with a continuous-time first-order autocorrelation structure for each null model.

To determine whether blue whale feeding locations are significantly different from those of non-selecting individuals (random site selection model), we used the adehabitatLT package (v. 0.4.19, [83]) to create 10 randomized, simulated tracks for each individual. The simulated tracks were implemented as a correlated random walk (CRW) [84] and were parameterized by the scaling parameter (*h*) and concentration parameter of turning angles (*r*) for each individual, using random starting points from within the minimum convex polygon of each deployment. Time-matched FTLE values were extracted for each simulated location. None of the simulations has points on land, and all simulated tracks use the same timestamps and feeding and non-feeding designation as their corresponding deployments to control for the temporal autocorrelation of the data as well as to better compare to the real whale data.

We also explored the temporal persistence of blue whale feeding locations and FTLE using time-shifted tracks. We shifted the timestamps of the movement data-stream for each individual forward in time by 24, 48, 96 and 192 h and extracted the time-matched FTLE value for each location to quantify the temporal persistence. All locations are identical to the real whale data and retain the same feeding designation. We hypothesized that if FTLE features are location-specific and persistent for multiple days, we would observe the same relationship across all time-shifted models and the real data.

#### (iii) Feeding rate analysis

We applied a hidden Markov model (HMM) using data-derived feeding states to estimate state probabilities in relation to FTLE and to assess the influence of FTLE on blue whale feeding rates [85]. These models take advantage of the inherent serial autocorrelation of animal tag data to determine the probability of switching between discrete behavioural states. We normalized the feeding rate by percentile rank (FR_pct_) for each individual to account for inter-individual differences in feeding rates and used these to classify feeding rates into groups in a HMM. For this analysis, we split the normalized feeding rates at the 25th and 75th percentiles to create four states: non-feeding (FR_pct_ = 0%), light feeding (FR_pct_ ≤ 25%), moderate feeding (25% < FR_pct_ ≤ 75%) and heavy feeding (FR_pct_ ≥ 75%). Using these four states as the *a priori* known states of the model, we fit a HMM using the moveHMM package (v. 1.7, [86]). We use a supervised learning approach [87] that summarizes the effect of FTLE on modelled behavioural transition probabilities to calculate the stationary probabilities of each state. These represent the estimated probability of being in each state as a function of FTLE [86]. We also fit a two-state (feeding and non-feeding) HMM to corroborate the GLMM results of the feeding site selection model.

To account for differences in tag deployment lengths, we used the weighted distribution of feeding rates for all animals to estimate the overall feeding rate in relation to FTLE of an average blue whale in our study. Weights were assigned such that the distribution of feeding rates for each of the 10 individuals counted evenly (see electronic supplemental material):

$${\mathrm{weight}}_{\mathrm{individual}}=\frac{1}{\mathrm{no}.\;\mathrm{feeding}\;{\mathrm{dives}}_{\mathrm{individual}}\times \mathrm{no}.\;\mathrm{deployments}}$$

We split the weighted distribution into four states using the same percentiles as the FR_pct_ and calculated the mean feeding rate for each state. Using the stationary state probabilities of the HMM, we estimated the overall feeding rate across the range of FTLE values *i* = −1.25 to 1.5 and made the same calculations using the upper and lower confidence intervals of the model:

$$\begin{array}{rl} & \mathrm{estimated}\;\;\mathrm{feeding}\;{\mathrm{rate}}_{\left({\mathrm{FTLE}}_{i}\right)}\\ & \;=\;\overset{4}{\underset{\mathrm{state}=1}{\sum }}\;\mathrm{stationary}\;{\mathrm{probability}}_{{\mathrm{state}}_{{\mathrm{FTLE}}_{i}}\;}\times \mathrm{feeding}\;{\mathrm{rate}}_{\mathrm{state}} \end{array}$$

## 3. Results

We examined the movement and foraging behaviour of 10 blue whales along the California coast using high-resolution multi-sensor tags, with deployments spanning Northern, Central and Southern California over 5 years between the months of May and November. Visual inspection of hourly plots of FTLE and time-matched blue whale locations showed a qualitative association between blue whale movements and feeding behaviour in relation to areas of elevated FTLE (electronic supplementary material animation). Through our statistical analysis, we found significant associations between FTLE features and regional habitat selection, feeding site selection and feeding rates for blue whales based on the more than 1700 h of tag and FTLE data. We examine each of these associations below.

### (a) Regional habitat selection

**Figure 3.**
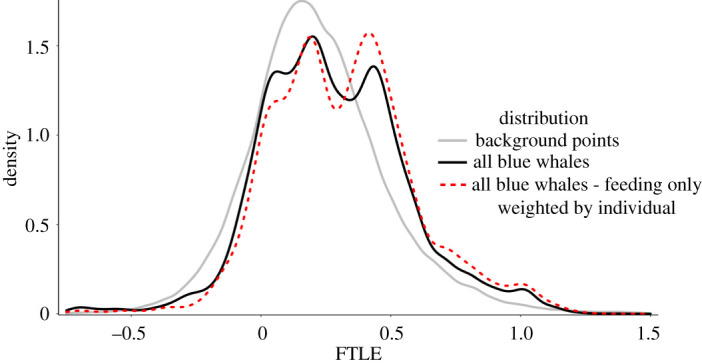
FTLE values were significantly greater at blue whale locations (black; *D* = 0.0617, *p* < 0.000001) than the background distribution (grey). Lines represent density distributions of FTLE for all animal locations (*n* = 7791, black), feeding locations only (*n* = 3875, red) and background samples (*n* = 77 910, grey); all distributions weighted by individual to account for varying deployment lengths.

When comparing the overall distribution of FTLE for all animal locations (individually weighted mean = 0.279) to that of the background samples (individually weighted mean = 0.221), we found that blue whale locations were co-located with significantly higher FTLE values (Kolmogorov–Smirnov test *D* = 0.0617, *p* < 0.000001, figure 3). Additionally, we found that when comparing each individual to background samples drawn from the corresponding regional spatial domain and time-period for each individual, FTLE values were significantly higher at whale locations than the background distributions for all 10 individuals (table 1).

### (b) Feeding site selection

From our high-resolution tag data, we identified blue whale feeding behaviour (i.e. a dive with one or more lunges) in 46 ± 14% of recorded dives. For blue whales in our study, an extended sequence without a 10 m dive was rare (0.5% of dives had a surface time greater than 60 min and 98% of dives with surface times greater than 20 min were not feeding dives) and greater than 95% of dive locations had a gap of two or fewer dives between them for all individuals. We used the presence or absence of feeding behaviour and location for each dive to examine blue whale feeding site selection and found that blue whales selected stronger FTLE features during feeding than non-feeding dives and more than would be expected from random feeding site selection.

**Figure 4.**
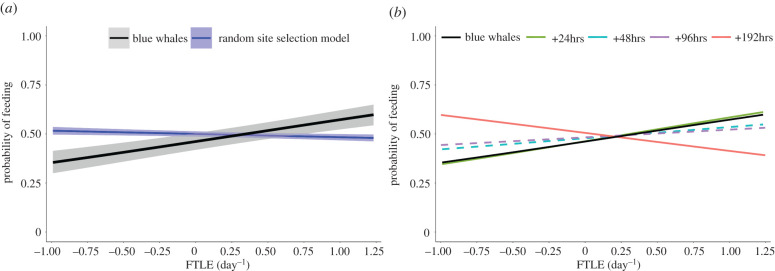
(*a*) The probability of feeding increased with FTLE (black with CI shaded, *n* = 10 individuals, 7791 dives, slope 0.45, *p* = 0.0025). The slope of the relationship was significantly greater than the CRW null model (blue with CI shaded, *n* = 100 simulated tracks, slope −0.066, *p* = 0.156). (*b*) The slope of the feeding rate–FTLE relationship decreased when tracks were time-shifted, with a greater decrease for larger time shifts. Dashed lines in (*b*) represent *p*-values greater than 0.05 (i.e. the predicted slope was not significantly different from 0).

FTLE positively influenced the probability of feeding for blue whales in our study (*n* = 10 individuals, 7791 dives; GLMM: slope 0.448, *p* = 0.0025). We also used a GLMM to test a hypothesis of no selection using CRW simulated tracks. The random site selection model produced a weak negative relationship between FTLE and the probability of feeding (*n* = 100 simulated tracks; GLMM: slope −0.066, *p* = 0.156, figure 4*a*). When we explored the temporal persistence of FTLE features, we found that the relationship between blue whale feeding site selection and FTLE did not persist for temporal shifts greater than 24 h. The +24 h model was significant, with a similar relationship to the real data (GLMM slope 0.49, *p* < 0.003). Temporal shifts of 48 and 96 h showed no relationship, while a shift of 192 h had a significant negative relationship (figure 4*b*; electronic supplementary material, table S1).

### (c) Influence on feeding rates

**Figure 5.**
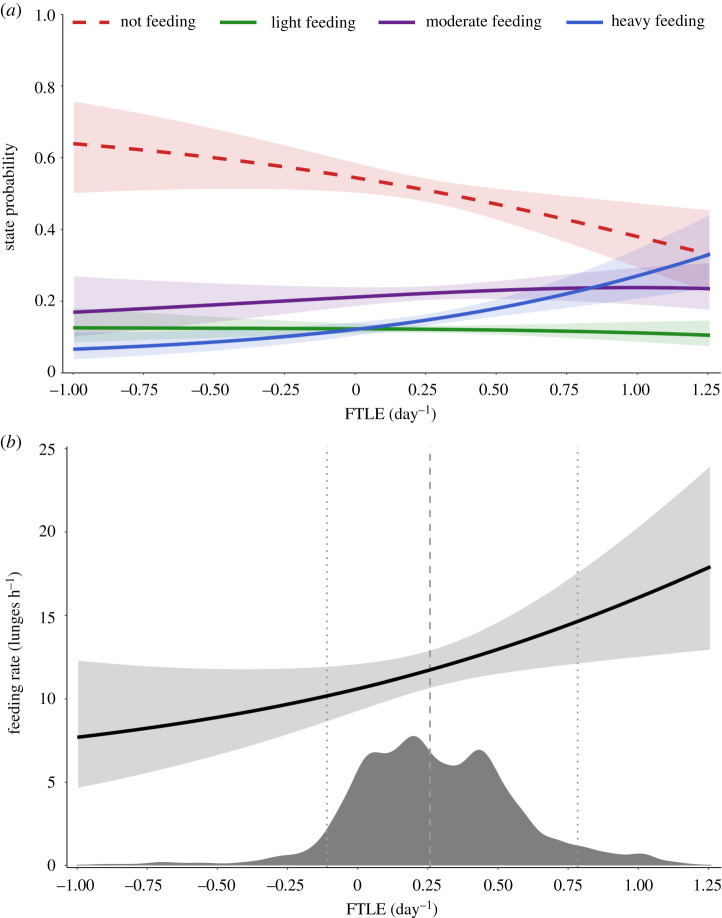
(*a*) The stationary state probabilities for the four-state feeding rate HMM; (*b*) the estimated overall feeding rates using the modelled stationary state probabilities in (*a*) and the mean feeding rates for each state for an average blue whale in our study; a histogram rug plot (dark grey) shows the distribution of encountered FTLE by all individuals in our study (weighted by individual) with vertical lines at the 5th, 50th and 95th percentiles.

Feeding intensity increased with FTLE strength. The mean dive-by-dive feeding rate (see methods) for all individuals was 23.8 ± 7.4 lunges h^−1^. When we calculated state probabilities from the normalized feeding rates for the four-state HMM (figure 5*a*), we found a negative relationship between FTLE and the non-feeding state, concordant with the relationship found in both the two-state (feeding and non-feeding) HMM and the feeding site selection analysis (electronic supplementary material, figure S1). The stationary probabilities of the three feeding states (light, moderate and heavy) all covaried with FTLE such that feeding intensity increased with FTLE strength (figure 5*a*). At low FTLE values, light and moderate feeding were more probable than heavy. As FTLE values increase, heavy and moderate feeding become more probable while light feeding less so. The mean feeding rates for the average blue whale's feeding distribution for each state (i.e. light, moderate and heavy feeding states) were 13.5, 23.9 and 34.1 lunges h^−1^, respectively (electronic supplementary material, figure S3). The overall feeding rate for an average blue whale in our study increased with FTLE (figure 4*b*). When modelling estimates at the 5th, 50th and 95th percentiles of the weighted distribution of encountered FTLE for all individuals (table 2), we found an overall increase of 43% in estimated feeding rate for an average blue whale in our study between the 5th and 95th percentiles (+15% between the 5th and 50th; +25% between the 50th and 95th).

**Table 2.** Model estimates at the 5^th^, 50^th^ and 95^th^ percentiles of the weighted distribution of encountered FTLE for all individuals. Estimated feeding rate is for the average blue whale in our study.

estimate	percentile encountered FTLE		
	5^th^	50^th^	95^th^
encountered FTLE (day^−1^)	−0.11	0.26	0.78
probability (not feeding)	0.56	0.51	0.42
probability (light feeding)	0.12	0.12	0.12
probability (moderate feeding)	0.21	0.22	0.24
probability (heavy feeding)	0.11	0.15	0.23
estimated feeding rate (lunges h^−1^)	10.43	11.98	14.95

## 4. Discussion

Our study reveals a consistent functional relationship between aggregative surface current features, regional and site-specific habitat selection, and feeding rates, in which blue whales disproportionately select and forage within dynamic aggregative features measured at hourly intervals across seasons, regions and years. As obligate krill feeders [23,25], blue whales' behavioural responses to patchy environments would predominantly be reflected in movement rather than prey-switching. We coupled contemporaneous measurements of blue whale movement and foraging behaviour with empirically derived submesoscale current features to evaluate blue whale foraging performance at fine spatio-temporal scales. These results build upon previous research (e.g. ([13,37–39]) that found aggregative surface features are important to cetaceans and other top predators’ foraging behaviour and support our hypothesis that submesoscale oceanic features influence blue whale foraging performance. The ability to detect individual feeding events is rare in biologging studies [88], and to our knowledge, this is the first study to link submesoscale oceanic features to predator feeding rates. This key development demonstrates that the association found in previous studies between energy gain and oceanic features at mesoscale spatial scales [3] is likely driven by submesoscale interactions.

Our study tracked 10 individuals in different seasons, regions and years for up to 18 days (mean 7.14 ± 4.98). Though our sample size of individuals was relatively small, the longevity of the deployments combined with the high-resolution behavioural and environmental data represents a substantial advance in our understanding of habitat selection processes in the dynamic marine environment. For this study system, coastal HF radar provided higher spatio-temporal resolution environmental data than satellites, without sacrificing synoptic coverage. Further, by drawing on previous research on balaenopterid foraging kinematics [19,28], we were able to directly observe feeding rates, rather than relying on more distal proxies for foraging performance such as patch residence time.

Our models indicate that the relationship between blue whale movement and surface current features deteriorates after 24 h, suggesting these features are ephemeral at timescales relevant to foraging decisions. We used FTLE as a proxy for these underlying oceanographic processes because it captures the time-dependent nature of this dynamic environment. If the aggregative surface current features measured here were geographically static, we would expect the observed relationship between foraging locations and FTLE to persist through time. While some aggregative surface current features may persist beyond the 24 h timescale, more research is needed to understand their formation and persistence in the environment.

The ecological links between surface dynamics and sub-surface habitat remain difficult to study [89], and the depth of influence of the surface features we describe is not precisely known. However, surface current features have been shown to reflect oceanographic processes that propagate through the water column, potentially to depths of over a kilometre [40], below the depth of blue whale foraging [90]. Prior work has shown associations between surface metrics and sub-surface foraging behaviour of deep-diving species such as fin whales [39], elephant seals [3] and both shallow and deep-diving penguins [13]. While surface current features and organisms such as krill may be disjointed during the day, diel vertical migration may further increase the importance of surface features when prey species forage during the night.

The covariance between blue whale feeding rates and FTLE strength (figure 4*b*) may indicate increased krill abundance or density in submesoscale aggregative ocean features. Prey patch density is particularly important to the foraging efficiency and survival of blue whales due to the high energetic cost of lunge feeding [28]. Previous research found that blue whales in the CCS increase feeding rates when encountering patches of higher prey density [49], and foraging decisions may be more influenced by patch density than total patch biomass [22]. In the CCS, most krill aggregations are short-lived (e.g. 2–10 days) and large, persistent krill aggregations are rare [44]. The short duration and fine-scale heterogeneity of these dense patches underscores (i) the importance of understanding intermediate and fine-scale biophysical relationships that drive these hotspots and (ii) the need for contemporaneous measures of predator movements at similar scales [42,91]. A recent study in Monterey Bay found that krill aggregate in response to changing wind regimes at scales similar to the measurements of this study (i.e. hours to days), but the mechanism for this phenomenon is not well understood [45]. Further research is needed to better understand how FTLE features and oceanographic conditions influence the distribution and density of krill in the CCS.

Consumer fitness relies on optimizing energy intake for allocation to survival, growth and reproduction [92,93]. Energy intake is modulated by several processes, primarily feeding rate and prey choice. Feeding rates exhibit functional responses to environmental heterogeneity, food density [94] and biotic interactions [95]. The effects of prey density and biotic interactions (e.g. interference competition and predator vigilance) on feeding rates have been well-studied, but environmental heterogeneity less so. In the terrestrial environment, for example, granivorous birds' feeding rates were negatively correlated with substrate complexity; i.e. higher in bare soil than crop stubble [96], whereas insectivorous birds’ feeding rates remained constant across a range of changing environmental conditions by switching from aerial to ground foraging tactics in inclement weather [97]. However, the marine environment is more spatio-temporally dynamic than most terrestrial ecosystems [98]. Fine-scale, ephemeral features revealed by FTLE, such as fronts and eddies, provide physical structure in a moving medium, aggregating energy, nutrients and biomass into dynamic patches. As a result, the spatio-temporal scales of marine predators' movements and foraging decisions differ qualitatively from their terrestrial counterparts [34,99]. The scales investigated in this study are therefore necessary for quantifying marine predators’ functional responses to environmental heterogeneity and developing a holistic understanding of how consumers optimize energy intake.

This study leverages advances in biologging (i.e. high-resolution tags) and remote sensing (i.e. HF radar) to provide insight at scales that were previously difficult to measure. Although other remote-sensing platforms—such as unoccupied aerial vehicles—can capture both behaviour and the environment at fine-spatial scales [100], we show that the combination of biologging and HF radar can provide a broader scope of high-resolution behavioural detail and synoptic oceanographic coverage. These results provide further evidence that fine- and intermediate-scale processes are important to pelagic predators and suggest that predator behaviour could help identify gradients in habitat quality (e.g. ecotones) and elucidate the timescales of patchiness. Here we expand upon existing techniques (e.g. satellite altimetry) to highlight the efficacy of using a HF radar system to identify submesoscale features, which can be used to predict heterogeneously distributed prey resources and critical habitat in near real time. The heavy use of fine-scale, dynamic features by foraging blue whales underscores the need to take dynamic habitat features into account when designating critical habitat and may help inform strategies to mitigate the impacts of human activities for the species.

## Data Availability

HF radar data were provided by the U.S. Integrated Ocean Observing System (IOOS) High Frequency Radar Network (HFRNet) and accessed through the Coastal Observing R&D Center (CORDC). The code used in data processing and analyses is available at the following public repository: https://github.com/physalus/Blue-Whales-and-Lagrangian-Features. Code and processed data have been archived at: https://doi.org/10.5281/zenodo.5904196. Raw blue whale tag data and surface current data have been deposited at Stanford University's digital repository: https://purl.stanford.edu/kn066mv7984. Electronic supplementary material is available online [101].
